# Post-rape medicolegal service provision and policy in East Africa: a scoping review protocol

**DOI:** 10.1186/s13643-021-01613-9

**Published:** 2021-02-24

**Authors:** Sarah Rockowitz, Heather Flowe, Caroline Bradbury-Jones

**Affiliations:** 1 School of Psychology, University of Birmingham, 52 Pritchatts Road, Birmingham, B15 2SA UK; 2 School of Nursing, University of Birmingham, Birmingham, B15 2TT UK

**Keywords:** Sexual violence, East Africa, Policy, Service provision, Scoping review, Rape

## Abstract

**Background:**

Sexual and gender-based violence (SGBV) is an epidemic that continues to affect both men and women in East Africa. Despite the high prevalence of SGBV in this region, sexual offense policies are often unclear, poorly enforced, or completely lacking. When policies do exist practitioners who assist survivors in the aftermath of the violation often are unaware of them, or may not implement them for a host of reasons (e.g., culture, personal beliefs, and resource limitations). This scoping review seeks to evaluate the literature on existing sexual offense policies in East Africa and understand the consequences of its implementation, or lack thereof, on a survivor’s justice and medical and psychological wellbeing.

**Methods:**

This scoping review will be guided by the amended Arksey and O’Malley framework recommendations (Levac et al., Implementation Science. 2010) and the 2015 Joanna Briggs Institute guidelines (Peters et al., Joanna Briggs Institute Reviewer’s Manual, 2020). The results will be presented using the adapted Preferred Reporting Items for Systematic Reviews and Meta-Analysis: Extension for Scoping Reviews chart (PRISMA-ScR). The search strategy for this scoping review will include entering search terms into electronic databases, including PubMed, SCOPUS, CINAHL Plus, The British Library, and Web of Science. A “cited by” search will be conducted, which will also include entering references from the reference lists from other articles. Grey literature will be included in the review, which will be identified through searching individual country’s government websites, and other websites, such as the World Health Organization and the United Nations Human Rights Council. All references will be exported to Endnote library. Two independent reviewers will screen titles, abstracts, and full articles. Thematic analysis will be used to evaluate the included articles.

**Discussion:**

Understanding the legal and regulatory context of SGBV in East Africa and its associations with service provision will generate knowledge on implications for wellbeing. This information can be used to evaluate potential human rights violations and inform future policy.

**Systematic review registration:**

Open Science Framework https://osf.io/vh3gm

## Background

Sexual and gender-based violence (SGBV) is defined by the UN as an act perpetrated against a person’s will that is based on societal gender norms between males and females [1]. These acts may lead to physical, mental, or sexual harm or suffering, and include rape, forced or early marriage, or other forms of violence such as psychological abuse from both intimate partners and non-partners [2, 3]. The World Health Organization (WHO) estimates that 1 in 3 women globally will experience some form of physical and/or sexual violence in their lifetime. East Africa’s prevalence is often higher, with a 59% lifetime prevalence of sexual violence by an intimate partner in Ethiopia province, 47% prevalence of lifetime physical violence in Tanzania province, and 50% prevalence of lifetime intimate partner physical and/or sexual violence in Uganda [4, 5]. Sub-Saharan Africa also has some of the highest rates of child marriage in the world, with almost four out of 10 young and adolescent girls being married before the age of 18 [6].

SGBV is considered both a human rights violation and a public health issue that concerns all sectors of society [4]. The frequent interaction between sexual violence survivors and the health, justice, and social service sectors indicate that the strength of a country’s service infrastructure can have lasting effects on a survivor’s wellbeing. Furthermore, the lack of adequate medical care and poor handling of legal cases can be seen as further human rights violations on top of the assault itself. Although some studies exist evaluating different care models across the region, little work has been undertaken examining this issue from a policy and human rights perspective.

The effects of SGBV can include depression, poor reproductive health outcomes, alcohol and substance use, and even death [4, 7, 8]. Besides having personal effects on survivors, SGBV can have serious consequences for a country’s economy. Research done in Kenya has found that the overall loss to the economy due to SGBV could be as high as 10% when accounting for the resources provided for treatment, litigation, and other related activities [9]. In Uganda, the cost of the after effects of domestic violence have been estimated at UGX 22 billion (4.7 million GBP) annually for those seeking care and UGX 56 billion (12 million GBP) annually for providers and duty bearers [10]. Globally, the cost of violence against women could amount to around 2% of the global gross domestic product [11].

SGBV is a topic that has been at the forefront of many global human rights discussions and agreements. The Maputo Protocol encourages states to take measures to enact and enforce laws to prohibit all forms of violence against women and Sustainable Development Goal #5 promotes implementing new legal frameworks to aid in the eradication of harmful practices targeted at women [12, 13]. Despite the existence of both of these frameworks, as well as other international human rights treaties and regulations, SGBV continues to be a pressing issue in East Africa.

Despite East Africa’s high rates of SGBV, there is not a comprehensive source that compares policies and regulations throughout the region. Moreover, there are no evaluations of how the countries are taking steps to improve post-rape service provision. Countries in East Africa are in many different stages of development, stability, and international treaty participation. In fact, in East Africa all countries except Eritrea, Somalia, and Burundi have both signed and ratified the Maputo Protocol [12].

This scoping review seeks to examine and compare existing policies and/or regulations concerning gender-based violence in East Africa. It will also determine the nature of post-rape service provision in each country, assess it in light of the country’s policies, and then evaluate it from a human rights lens. The findings from this research will enable the researchers to examine the type of policies that are present throughout East Africa and evaluate their compliance with signed treaties and protocols. The findings will also allow the researchers to understand further the infrastructural barriers that may be prohibiting countries from instituting effective post-rape care services.

## Methodology

This scoping review protocol is registered and published in the Open Science Framework platform (registration ID: https://osf.io/vh3gm)

This scoping review will be conducted following the Arksey and O’Malley framework and further aided by the Levac et al. recommendations and Joanna Briggs Institute Guidelines [14–16]. The steps of the framework are as follows:

- Identify the research question
- Identify relevant studies
- Select studies
- Chart the data
- Collate, summarize, and report the results.

The write up of this protocol will then be guided by the PRISMA-ScR checklist [17].

### Step 1: Identifying the research question

The primary research question of this study is:

What are the existing regulations/policies regarding SGBV across East Africa and how do they affect the delivery of post-rape care services?

The sub-questions of this study are:

i. To what extent are current service provision models compliant with human rights law?
ii. How do duty bearers’ personal beliefs and practices affect their compliance with law and policy obligations?
iii. How well do the countries’ policies align with international treaties (if they have signed them) concerning violence against women?
iv. What are the key gaps in policies that are allowing continued human rights violations and inadequate service provision to occur?
v. What are existing gaps in the literature and what further research needs to be done?

### Step 2: Identifying relevant studies

This study will use a comprehensive search strategy, based on the Population, Concept, and Context (PCC) model from the Joanna Briggs Institute in order to identify relevant literature [15]. This model is an adapted version of the PICO (population, intervention, comparison, outcome) model, but is better suited for scoping reviews as there is no intervention or specific outcome that needs to be stated [15].

#### Inclusion criteria

- Population—Survivors of sexual and gender-based violence, government institutions, policy officials
- Concept—Full-text articles with a focus on post-rape service provision, policy documents pertaining to sexual violence, international treaties
- Context—Research on East Africa (Burundi, Comoros, Djibouti, Eritrea, Ethiopia, Kenya, Madagascar, Malawi, Mauritius, Mayotte, Mozambique, Reunion, Rwanda, Seychelles, Somalia, South Sudan, Tanzania, Uganda, Zambia, Zimbabwe), international human rights treaties, no date restrictions on policy documents

#### Exclusion criteria

- Studies of contexts other than East Africa
- Studies that are about other violations besides SGBV
- Studies published before 2000
- Policy documents not pertaining to East Africa

#### Identifying relevant studies

Research articles from peer-reviewed journals, review articles, and grey literature such as policy documents or international treaty documents will be included in this study. The electronic search process for this paper will follow the Joanna Briggs three-step strategy for scoping reviews, which includes an initial limited search of two online databases, followed by an analysis of relevant text words in the titles and abstracts of papers, and finally, a search of reference lists from identified reports and articles for additional sources [15].

Databases that will be included in the scoping review include PubMed, SCOPUS, CINAHL Plus, The British Library, and Web of Science. Government websites from each country will also be searched for policy documents and other forms of relevant grey literature. Once papers from these sources are gathered, a “cited by” search will also be conducted, as will a search using the reference lists of included articles.

This search will be limited to research and policy documents that are available in English. Although this may impact the number of documents available, it will also help ensure accuracy as translation services may be poor and it is important in this research to understand policy documents correctly. There is no time restriction on policy documents, and academic articles will be from 2000 onwards.

### Step 3: Study selection

All literature search results will be uploaded to Endnote X9 software for collation and duplicate removal. Once duplicates are removed, titles and abstracts will be screened and any studies or grey literature that do not address the research questions will be excluded. This database will then be shared with two independent reviewers who will screen abstracts and full articles with guidance from the inclusion criteria, and finally, copies of relevant papers will be kept for data extraction.

**Fig. 1:**
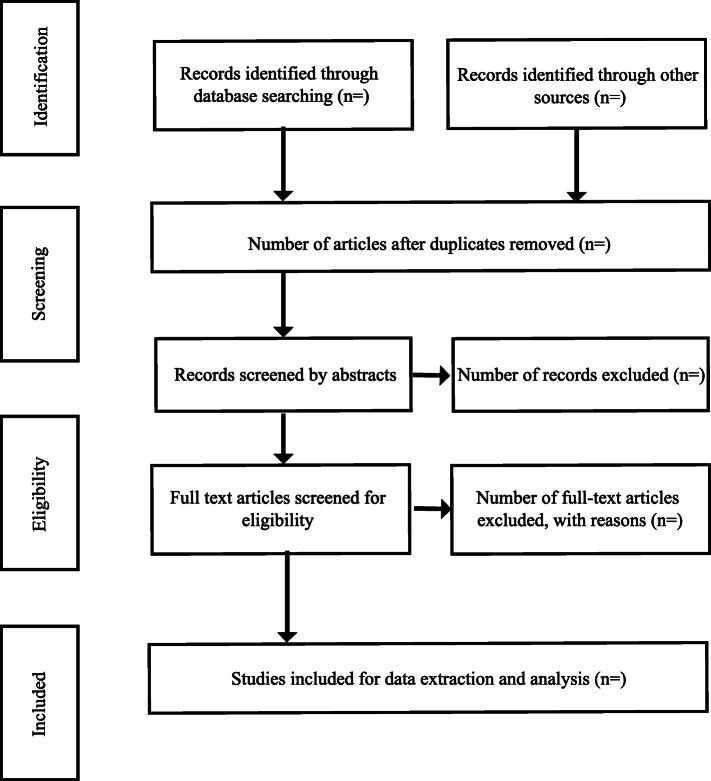
Example of PRISMA ScR chart. Source: Preferred Reporting Items for Systematic Reviews and Meta-Analyses: the PRISMA statement [17]

Once this review is completed, an adapted version of the PRISMA flow diagram (Fig. 1) will be used to report final numbers in the subsequent study publication.

### Step 4: Data charting

A standardized data-charting sheet will be developed to allow the researchers to keep and organize key pieces of information from the articles. This chart may be altered as more data is managed as per Levac et al.’s acknowledgement that data synthesis is an iterative process [14]. By organizing the data as such, a descriptive summary of each article or piece of grey literature will be easily accessible and will allow for better data management by the researchers. Information in this chart will include bibliographic details, study location, type of publication, and key findings (Table 1).

**Table 1:** Data extraction tool

Author and year of publication	
Type of publication	
Country	
Study design	
Study setting	
Key findings	
Conclusion	

### Step 5: Collating, summarizing, and reporting the results

Data will be extracted from the literature using content and thematic analysis by the researcher and two independent reviewers, which will then enable the researchers to develop a narrative account of the findings. The themes will be structured around types of service provision in each country, availability of policy pertaining to SGBV, each country’s compliance with their own policies, and how a country’s policies and regulations relate to signed international treaties. To aid the coding and data synthesis, NVivo version 12 will be used.

## Discussion

As far as we are aware, this scoping review will be the first to compare policies on SGBV through East Africa and evaluate how the policies translate to in-country service provision. Preliminary searches of SCOPUS, Google Scholar, and Prospero largely yielded results concerning intimate partner violence (IPV) in Africa and potential interventions [18, 19], but no papers were found that were not intervention-focused or based on a smaller population, i.e. pregnant women. It will also compare how each country’s policies may or may not be aligned with international development goals and human rights instruments, which will be beneficial in providing evidence for why a country may need to alter or improve their method of service provision for SGBV survivors. With worldwide focus on SGBV increasing in recent years, especially concerning the #MeToo movement and Sustainable Development Goal 5, this review is a timely and important way to synthesize knowledge for a part of the world where SGBV persists in some of its highest numbers globally. The researchers hope that this review will assist in gathering evidence for how sexual assault care provision throughout the region of East Africa can and should be improved, and additionally, that it will inform policy makers in the future. Findings from this review will be disseminated through publication in journals, presented at conferences, and used to inform future research.

## Data Availability

All data generated during this study will be included in the published scoping review and will also be made available upon request.

## Abbreviations

PRISMA-ScR: Preferred Reporting Items for Systematic Reviews and Meta-Analyses extension for scoping reviews
SGBV: Sexual and gender-based violence
UN: United Nations
WHO: World Health Organization
